# Platelet count can predict the grade of esophageal varices in cirrhotic patients: a cross-sectional study

**DOI:** 10.12688/f1000research.28005.1

**Published:** 2021-02-11

**Authors:** Anum Afsar, Muhammad Nadeem, Syed Asim Ali Shah, Huma Hussain, Aysha Rani, Sadaf Ghaffar

**Affiliations:** 1 POF Hospital, Wah Cantt, Punjab, Pakistan; 2 Department of Medicine, Poonch Medical College, Rawalakot, Rawalakot, Pakistan; 3 Department of Medicine, Wah Medical College, Wah Cantt, Punjab, Pakistan

**Keywords:** Cirrhosis, Platelet, Varices

## Abstract

**Background: **Bleeding from esophageal varices is a life-threatening complication in cirrhosis. Screening endoscopy is recommended in cirrhotic patients to identify patients at risk of variceal hemorrhage, but this is an invasive procedure and has limitations. Therefore, thrombocytopenia has been proposed to predict the existence and grade of esophageal varices. The aim of the current study was to determine a correlation between platelet count and grades of esophageal varices in patients with liver cirrhosis.

**Methods:** This cross-sectional study was conducted at the POF Hospital, Wah Cantt from 1 ^st^ October, 2017 to 30 ^th^ May, 2018. Newly diagnosed cases of cirrhosis having varices of any grade on endoscopy were included. Endoscopic findings of patients were standardized using Paquet grading system. On the basis of platelet count, patients were divided into four subgroups. Platelet count groups were correlated with grading of esophageal varices using Spearman rank correlations. Chi Square test was used to see association between the platelet count and grade of esophageal varices.

**Results: **110 patients were included in the study, 55.5% (n=61) were male. Mean age of the patients was 59.89±9.01 years. Platelet count was <50,000/uL in 35.5% patients, 50,000-99,000/uL in 26.4%, 100,000-150000 in 12.7%, and >150,000/uL in 25.5% patients. Grade I esophageal varices were found in 23.6% of patients, whereas grade II, III and IV were found in 24.5%, 33.6% and 18.2% of patients, respectively. Mean platelet count was 213884.62/mm ^3^ in patients with grade I varices, whereas it was 119518.52/mm ^3^, 58386.49/mm ^3^ and 21600.00/mm ^3^ in patients with grade II, III and IV varices, respectively (p=<0.0001). A significant negative correlation between platelet count and grades of esophageal varices was found (p<0.001).

**Conclusion: **Platelet count can predict the grade of esophageal varices in cirrhotic patients. There is significant negative correlation between platelet count and grades of esophageal varices.

## Introduction

Portal hypertension is a common complication of liver cirrhosis which can lead to esophageal varices that may rupture and bleed ^1^. Bleeding from esophageal varices is life threatening and comprises 10% of all cases of upper gastrointestinal (GI) bleeding ^2^. Esophageal varices are present in approximately one third of patients at diagnosis of cirrhosis and incidence increases to 90% in 10 years ^3^. The rate of progression from small to large varices is estimated to be 8–10% per year and the annual rate of esophageal hemorrhage is 5% for small varices and 15% for large varices ^3, 4^. Screening endoscopy is therefore recommended for early detection of esophageal varices and grading of varices in cirrhotic patients to identify patients with risk of variceal hemorrhage and administer prophylactic treatment if required ^5^. Screening endoscopy is however an invasive procedure and has limitations ^6^.

Diagnosis and grading varices by endoscopy is operator dependent. This approach also places a heavy burden upon endoscopy units and repeated testing over time may affect patient compliance, and endoscopic screening also increases the associated health care costs ^7^. Due to these problems regarding endoscopy, some noninvasive means have been proposed for prediction of esophageal varices and their grades in order to restrict endoscopy to the population with high risk of variceal bleeding ^7^. Various clinical and biochemical predictors have been studied to predict the existence and grade of esophageal varices in cirrhotic patients ^8^.

Thrombocytopenia (platelet count <150,000 /uL) is one such predicator. It is found in approximately 64–76% of patients with portal hypertension and cirrhosis ^9^. The pathogenesis of thrombocytopenia in cirrhosis is multi-factorial and includes decreased thrombopoietin production, sequestration of platelets in spleen and direct myelosuppression due to hepatitis C virus ^10^. As thrombocytopenia and esophageal varices are common findings in cirrhosis, and portal hypertension is associated with both of these findings, some studies have been conducted to see the relation of thrombocytopenia as a non-invasive marker with grades of esophageal varices ^10, 11^. It was found in different studies that platelet count was inversely correlated with grades of esophageal varices ^11^. Large esophageal varices also appeared to have a lower platelet count in a study conducted at Shanghai East Hospital ^4^

In resource poor developing countries, endoscopy of every cirrhotic patient to grade the esophageal varices to select the patients for prophylactic therapy is not possible due to the limited number of endoscopes and endoscopists in government hospitals. We need to identify non-invasive and reliable markers to predict the grades of esophageal varices in our population in Pakistan, so that endoscopists can select the patients at increased risk of bleeding for endoscopy on priority basis. Therefore, we conducted this study to determine correlation between platelet count and grades of esophageal varices in our population and thus assess the possibility of using platelet count to predict the grades of esophageal varices.

## Methods

### Study design and participants

This cross-sectional descriptive study was conducted at the Department of Medicine, POF Hospital, Wah Cantt, Pakistan from 1 ^st^ October, 2017 to 30 ^th^ May, 2018. Newly diagnosed cases of cirrhosis having varices of any grade on endoscopy, irrespective of the cause of cirrhosis, with or without ascites and splenomegaly, from 18 to 70 years were included in the study. Diagnosis of cirrhosis was made using data obtained from clinical findings, laboratory investigations and ultrasonographic findings of the liver. Patients with hematological disorders, portal vein thrombosis, on B-blocker prophylactic therapy and those who had undergone endoscopic band ligation or sclerotherapy were excluded. 

The sample size calculated was 110 using sample size calculator n = (Z ^2^ × P × (1 - P))/d ^2^ for cross sectional studies, taking a confidence level 95%. In total, 110 patients were included by consecutive sampling.

Informed written consent was taken from patient or relative wherever relevant (consent was taken from relatives in patients with hepatic encephalopathy). Ethical approval was obtained from POF Hospital Research Ethics Committee before the start of the study (vide letter no. POFH/ERC/99053/03).

### Data collection

Endoscopy was performed by the same endoscopist using the Olympus GIF type Q260 endoscope. All patients were kept NPO for 8 hours before endoscopy and the endoscopic findings of the patients were standardized using the Paquet grading system for esophageal varices dividing esophageal varices by grades I to IV ^12^. Blood samples of all patients were taken by the staff nurse before endoscopy and sent to the laboratory of POF Hospital on the same day to calculate platelet count. Platelet count was calculated by automatic hematology analyzer (Sysmex XN-1000) and confirmed by manual method (small volume of whole blood was treated with RBC lysing reagent ammonium oxalate, was put in hemocytometer and platelet counting was done by hematologist using phase contrast light microscopy). All details including demographic details, endoscopic findings and platelet count were obtained from medical records of the selected patients. On the basis of platelet count, patients were divided into four subgroups: group 1,platelet count <50,000/uL; group 2,platelet count 50,000–99000 /uL; group 3,platelet count 100,000–150,000/uL; and group 4,normal platelet count >150,000/uL.

### Data analysis

All data was entered and analyzed using SPSS version 19.0. Mean and standard deviation was calculated for quantitative data, such as age. Frequency and percentage was calculated for gender, platelet count and grade of esophageal varices. Platelet count group was correlated with grading of esophageal varices using Spearman rank correlations. Chi square test was used to calculate the association between platelet count and grade of esophageal varices. P-value ≤0.05 was considered significant.

## Results

A total of 110 patients were included in the study, 55.5% (n=61) were male and 44.5% (n=49) were female. Mean age of the patients was 59.89±9.01 years. Platelet count was <50,000 /uL in 35.5% (n=39) of patients, 50,000–99,000/uL in 26.4% (n=29), 100,000–150000 in 12.7% (n=14), and >150,000/uL in 25.5% (n=28) patients. Grade I esophageal varices were found in 23.6% (n=26) patients, whereas grade II, grade III and grade IV were found in 24.5% (n=27), 33.6 (n=37) and 18.2% (n=20) of patients, respectively.

A significant association was found between the platelet count groups and grades of esophageal varices; groups with lower platelet counts had high-grade varices, while groups with higher platelet counts had low-grade varices ( Table 1).

**Table 1.** Association of platelet count group with grades of esophageal varices.

Platelet count		Paquet variceal grade				Total	p-value
		I	II	III	IV		
<50,000/uL	Group 1	2	3	16	18	39	<0.001
50,000–99,000/uL	Group 2	1	9	17	2	29	
100,000–150000/uL	Group 3	1	9	4	0	14	
> 150,000/uL	Group 4	22	6	0	0	28	

Spearman’s correlation showed a significant negative correlation between platelet count and grades of esophageal varices ( Table 2).

**Table 2.** Correlation of platelet count with grades of esophageal varices.

			Platelet count	Grades of esophageal varices
**Spearman's Rho**	Platelet count	Correlation coefficient	1.000	-0.783
		p-value	/	<0.001
	Paquet variceal grade	Correlation coefficient	-0.783	1.000
		p-value	<0.001	/

Mean platelet count was also significantly lower in patients with grade IV varices as compared to patients with grade I varices (p=0.0001; Table 3).

**Table 3.** Association of mean platelet count with Paquet variceal grade.

Paquet variceal grade	Mean platelet count (mm ^3^)	Std. deviation (mm ^3^)	N	p-value
I	213884.62	86434.867	26	<0.001
II	119518.52	68027.919	27	
III	58386.49	34188.433	37	
IV	21600.00	16161.683	20	

## Discussion

Our results showed that there is a significant negative correlation between the platelet count and grades of esophageal varices. Patients with a lower platelet count had varices of higher grades. In group 1 patients (platelet count <50,000//uL; n=39), 18 and 16 patients were found to have grade IV and grade III varices, respectively, as compared to only 2 and 3 patients having grade I and II varices, respectively. In contrast, in group 4 patients (platelet count >150,000/uL; n=28), 22 patients had grade I varices and no patients had grade III or IV varices. This suggests that platelet count can be used as a predictor of grades of esophageal varices without frequent upper GI endoscopy.

In our study, 55.5% (n=61) patients were male and 44.5% (n=49) were female. Studies have shown that the incidence of cirrhosis is lower in females as compared to males due to slower progression of fibrosis ^13^. In a study conducted in Taiwan, 71% cirrhotic patients were male ^14^. These findings are consistent with our findings of male predominance. Another study also supports our findings, as it shows that men had a higher incidence of cirrhosis in all age groups as compared to women of the same age group ^15^. A local study also showed that 64% of cirrhosis patients were men ^16^.

Mean age in our study was 59.89±9 years. Although cirrhosis of the liver can develop at any age, it is usually common in old age with a higher mean age of incidence ^15^. Mean age was found to be 40.5 years in cirrhosis patients in a study conducted by Devrajani *et al.* ^16^. This is comparable with our results. A study conducted in China showed a mean age of 50.29±7.03 years in cirrhosis patients ^17^, which is similar to a mean age of 54.39±7.46 years in a study conducted by Abd-Elsalam *et al.* ^10^, almost the same mean age as found in our study.

A significant inverse correlation was found between platelet count and grades of esophageal varices in our study. Lower platelet count was associated with high varices and vice versa. Platelet count is low in advanced cirrhosis; similarly, large varices are associated with advance cirrhosis. Many studies conducted previously to see the relationship of platelet count with esophageal varices concluded that lower platelet count is associated with large varices. For example, a study conducted by Abbasi *et al.* shows that platelet count is inversely related with esophageal varices, supporting our findings ^11^. Similarly, a study conducted in India also had the same results; lower platelet count was significantly associated with large varices ^18^.

Mean platelet count was significantly lower in patients with grade III (58386.49±34188.43mm ^3^) and IV (21600.00±16161.68mm ^3^) esophageal varices as compared to grade I (213884.62±86434.86mm ^3^) and II (119518.52±68027.91mm ^3^) varices in our study. These results are comparable with a study conducted in Egypt, where platelet count was significantly lower in patients with grades II and III (165.2±13.0 and 60.3±14.1 mm ^3^, respectively) than in patients with grade I (100.5±19.8mm ^3^) ^19^. According to these findings, platelet count can be used to identify patients who may have large varices and need prophylactic endoscopic treatment to prevent upper GI bleeding rather than doing endoscopy in every patient with cirrhosis. Platelet count is not only noninvasive, it is also inexpensive, resource effective, does not need special expertise, and is easily available.

Our study has some limitations: it was a single center study including all patients of cirrhosis irrespective of the cause; we only used platelet count to predict the grade of varices, some studies have shown that platelet count and spleen size ratio is more accurate in predicting the size and grade of varices. In future, multicentre studies with specific cause of cirrhosis and using platelet count and spleen size ratio are suggested.

## Conclusion

There is significant negative correlation between platelet count and grades of esophageal varices. Low platelet count is associated with higher grades of esophageal varices. This suggest that platelet count can be used as a predictor to predict the grade of esophageal varices. 

## Data availability

### Underlying data

Figshare: Platelet count can predict the grade of esophageal varices in cirrhotic patients, https://doi.org/10.6084/m9.figshare.13379939.v1 ^20^.

Data are available under the terms of the Creative Commons Attribution 4.0 International license (CC-BY 4.0).
