# Supplementary material for: Platelet count can predict the grade of esophageal varices in cirrhotic patients: a cross-sectional study
Source: F1000Res. 2021 Nov 24;10:101. Originally published 2021 Feb 11. [Version 2] doi: 10.12688/f1000research.28005.2 (PMC8729194; doi:10.12688/f1000research.28005.2)
Supplement: Peer review history

# Peer review: Reviewer response for version 2

**DOI:** 10.5256/f1000research.79401.r101099

**Published:** 2022-01-04

**Authors:** Muhammad Asim Anwar

**Affiliations:** 1 Pakistan Atomic Energy Commission, Islamabad, Pakistan; 2 PAEC General Hospital, Islamabad, Pakistan

i have no further comments to make.

Is the work clearly and accurately presented and does it cite the current literature?

Yes

If applicable, is the statistical analysis and its interpretation appropriate?

I cannot comment. A qualified statistician is required.

Are all the source data underlying the results available to ensure full reproducibility?

Yes

Is the study design appropriate and is the work technically sound?

Yes

Are the conclusions drawn adequately supported by the results?

Yes

Are sufficient details of methods and analysis provided to allow replication by others?

Yes

Reviewer Expertise:

internal medicine/gastroenterology/hepatology

I confirm that I have read this submission and believe that I have an appropriate level of expertise to confirm that it is of an acceptable scientific standard.

# Peer review: Reviewer response for version 2

**DOI:** 10.5256/f1000research.79401.r101100

**Published:** 2022-01-04

**Authors:** Alexandru Constantin Moldoveanu

**Affiliations:** 1 Gastroenterology and Internal Medicine II Clinic - University Emergency Hospital Bucharest, Carol Davila University of Medicine and Pharmacy, Bucharest, Romania

The second version of the article adds more context to the research, including limitations of the study which will be useful for future research in this field. A more thorough statistical analysis was performed, which further supports the conclusions of the article.

Is the work clearly and accurately presented and does it cite the current literature?

Yes

If applicable, is the statistical analysis and its interpretation appropriate?

Partly

Are all the source data underlying the results available to ensure full reproducibility?

Yes

Is the study design appropriate and is the work technically sound?

Yes

Are the conclusions drawn adequately supported by the results?

Partly

Are sufficient details of methods and analysis provided to allow replication by others?

Yes

Reviewer Expertise:

Gastroenterology, Gastrointestinal Endoscopy, Hepatology

I confirm that I have read this submission and believe that I have an appropriate level of expertise to confirm that it is of an acceptable scientific standard.

# Peer review: Reviewer response for version 1

**DOI:** 10.5256/f1000research.30974.r90353

**Published:** 2021-08-20

**Authors:** Alexandru Constantin Moldoveanu

**Affiliations:** 1 Gastroenterology and Internal Medicine II Clinic - University Emergency Hospital Bucharest, Carol Davila University of Medicine and Pharmacy, Bucharest, Romania

Esophageal varices are a frequent complication of cirrhosis and can be a life-threatening complication if ruptured. The risk of variceal bleeding is, however, not equal between patients, and the current gold standard method for assessing bleeding risk is upper gastrointestinal endoscopy - an invasive procedure, that is not easily tolerated by the patient. There are multiple non-invasive methods that were proposed over the years as either an alternative to endoscopy or as a method of reducing the number of endoscopies by performing them selectively only in patients with a high probability of also requiring endoscopic treatment.

 The authors’ study presents a very simple parameter, thrombocyte count, as a non-invasive and very cost-effective alternative method of identifying patients with different grades of esophageal varices. Considering that both hypersplenism (and thrombocytopenia) and esophageal varices are results of portal hypertension, it appears to be a reasonable presumption that the two would be correlated.

 The results indeed show as a very good correlation between the size of varices and platelet count, in both group analysis as well as continuous variable analysis. What is most interesting to me is that the platelet count has a very good sensitivity (approximatively 93% based on the results) for predicting patients with large varices (grades III and IV) requiring prophylactic endoscopic treatment. No patient with normal thrombocytes has had large varices in this study, which suggests that these patients might not require diagnostic endoscopy.

 Regarding the statistical analysis methodology, I believe that the methods could be expanded upon. An analysis of variance type test between groups would probably reveal statistically significant results given the values presented. Another possible analysis method is classifying patients into two groups: 1. needing endoscopic treatment and 2. not needing endoscopic treatment, and plotting an ROC curve, which could be used to determine sensitivity and specificity for an optimal cut-off.

 One criticism that I could make is that imminent bleeding signs, such as red wale signs, were not assessed. While these signs appear more frequently in large varices, they have frequently been reported in grade 2 varices as well, making it more difficult to assess which patients do not require endoscopy using the grade of the varices alone.

 Another criticism of the article is that, while the conclusions are very interesting, the information is not entirely new. The Baveno VI consensus has already proposed thrombocyte count as one of the most important factors to determine which patients can be ruled out from screening endoscopy. The authors’ study still has practical value for medical centres where measurement of the other main parameter in the Baveno VI consensus, liver stiffness, is either unavailable or not sufficiently cost-effective.

Is the work clearly and accurately presented and does it cite the current literature?

Yes

If applicable, is the statistical analysis and its interpretation appropriate?

Partly

Are all the source data underlying the results available to ensure full reproducibility?

Yes

Is the study design appropriate and is the work technically sound?

Yes

Are the conclusions drawn adequately supported by the results?

Partly

Are sufficient details of methods and analysis provided to allow replication by others?

Yes

Reviewer Expertise:

Gastroenterology, Gastrointestinal Endoscopy, Hepatology

I confirm that I have read this submission and believe that I have an appropriate level of expertise to confirm that it is of an acceptable scientific standard, however I have significant reservations, as outlined above.

# Response

**Published:** 2021-11-11

**Authors:** Muhammad Nadeem

**Affiliations:** Poonch Medical College Rawalakot AJK, Pakistan

We would like to thank the reviewer for his valuable time and comments on our article; this will be very helpful for us in future research. We had made few changes as suggested by reviewer.

 1. We applied one way ANOVA (Analysis of Variance) test and added in results.

 2. Reviewer raised an important point regarding the imminent bleeding signs, which we missed during data collection. As it is not possible to include it in article (we do not have data), we had mentioned it in limitations of our study in revised version so researchers in future can include this point.

 Another concern of reviewer was “information is entirely not new”. We agree with this statement but most of the studies used other parameters as well along with platelet count like spleen diameter, liver stiffness as mentioned by reviewer also. We used only platelet count to predict the varices size which is valuable for centres in developing world where other facilities are not available or cost is a problem, this was the main purpose of the study and also appreciated by reviewer.

# Peer review: Reviewer response for version 1

**DOI:** 10.5256/f1000research.30974.r87611

**Published:** 2021-06-30

**Authors:** Muhammad Asim Anwar

**Affiliations:** 1 Pakistan Atomic Energy Commission, Islamabad, Pakistan; 2 PAEC General Hospital, Islamabad, Pakistan

In this cross sectional of the platelet count predictability of esophageal varix is important study for those countries where the availability of the endoscopy services are few and the burden of disease is high. You can also schedule the endoscopy procedure where the predictability of high grade varix is high. Methodology is fine and the results are also presented well. In the discussion authors should also give some details of other non invasive methods like platelet spleen index.

 In conclusion there is significant negative correlation between platelet count and grade of varix

Is the work clearly and accurately presented and does it cite the current literature?

Yes

If applicable, is the statistical analysis and its interpretation appropriate?

I cannot comment. A qualified statistician is required.

Are all the source data underlying the results available to ensure full reproducibility?

Yes

Is the study design appropriate and is the work technically sound?

Yes

Are the conclusions drawn adequately supported by the results?

Yes

Are sufficient details of methods and analysis provided to allow replication by others?

Yes

Reviewer Expertise:

internal medicine/gastroenterology/hepatology

I confirm that I have read this submission and believe that I have an appropriate level of expertise to confirm that it is of an acceptable scientific standard.

# Response

**Published:** 2021-11-11

**Authors:** Muhammad Nadeem

**Affiliations:** Poonch Medical College Rawalakot AJK, Pakistan

We would like to thank the reviewer for his valuable time and comments on our article; this will be very helpful for us in future research.

 We added the some details of other non invasive methods in discussion as advised by reviewer
